# Prophylactic Cranial Irradiation in Patients With Non-Small-Cell Lung Cancer: A Systematic Review and Meta-Analysis of Randomized Controlled Trials

**DOI:** 10.3389/fonc.2018.00115

**Published:** 2018-04-20

**Authors:** Karine A. Al Feghali, Rami A. Ballout, Assem M. Khamis, Elie A. Akl, Fady B. Geara

**Affiliations:** ^1^Department of Radiation Oncology, American University of Beirut Medical Center, Beirut, Lebanon; ^2^Faculty of Medicine, American University of Beirut, Beirut, Lebanon; ^3^Department of Diagnostic Radiology, American University of Beirut Medical Center, Beirut, Lebanon; ^4^Department of Internal Medicine, American University of Beirut Medical Center, Beirut, Lebanon

**Keywords:** systematic review, meta-analysis, non-small-cell lung cancer, prophylactic cranial irradiation, survival, metastasis, brain, lung cancer

## Abstract

**Background:**

We systematically reviewed the literature for trials addressing the efficacy of prophylactic cranial irradiation (PCI) in patients with non-small-cell lung cancer (NSCLC) treated with a curative intent.

**Methods:**

Randomized controlled trials (RCT) comparing PCI to no PCI in patients with NSCLC treated with a curative intent were eligible for inclusion. We searched EMBASE, MEDLINE, PubMed, and CENTRAL between 1946 and July 2016. We also received continual search alerts from PubMed through September 2017. Search terms included “non-small-cell lung carcinoma,” “cranial irradiation,” and “randomized controlled trials.” We conducted meta-analyses using random-effects models for relative measures of treatment effect for the incidence of brain metastasis, overall survival (OS), and disease-free survival (DFS). We used Parmar’s methodology to derive hazard ratios (HR) when not explicitly stated in RCTs. We narratively synthesized data for the impact of PCI on quality of life (QoL) and neurocognitive function (NCF). We assessed the quality of evidence using the Grading of Recommendations, Assessment, Development, and Evaluation methodology.

**Results:**

Out of 3,548 citations captured by the search strategy, we retained 8 papers and 1 abstract, reporting on 6 eligible trials. Patients who received PCI had a significant reduction in the risk of developing brain metastases as compared with patients who did not [relative risk (RR) = 0.37; 95% confidence interval (CI): 0.26–0.52; moderate quality evidence]. However, there was no OS benefit (HR = 1.08, 95% CI: 0.90–1.31; moderate quality evidence). Sensitivity analysis excluding older studies did not show substantively different findings. DFS was reported in the two most recent trials that included only stage III patients. There was significant improvement in DFS with PCI (HR = 0.67; 95% CI: 0.46–0.98; high quality evidence). Two studies that reported on QoL reported no statistically significant differences. There was no significant difference in NCF decline in the only study that reported on this outcome, except in immediate and delayed recall, as assessed by the Hopkins Verbal Learning Test.

**Conclusion:**

There is moderate quality evidence that the use of PCI in patients with NSCLC decreases the risk of brain metastases, but does not provide an OS benefit. However, data limited to stage III patients suggests that PCI improves DFS, with no effect on QoL.

## Introduction

### Rationale

Lung cancer is predicted to be the second most commonly diagnosed cancer in 2017 in the United States, second to breast cancer in women and prostate cancer in men (1). It is also the most common cause of cancer-related deaths in men and women in the United States, accounting for more than one-quarter of cancer-related deaths (1). Non-small-cell lung cancer (NSCLC) accounts for approximately 80–85% of lung cancer cases (2, 3).

The brain is a frequent site of metastasis in both small cell lung cancer (SCLC) and NSCLC. The advances of the past two decades in the use of multimodality therapy, effective systemic therapy, and optimization of radiation therapy (RT), have improved locoregional and systemic control of the cancer. However, these have also paradoxically led to an increase in the proportion of patients with brain metastases (4–7). The brain is indeed considered a sanctuary site, with the presence of the blood–brain barrier preventing the passage of most systemic treatments. As a matter of fact, the brain is reported across multiple studies to be the first site of failure after curative treatment in about 14–28% of patients with NSCLC (4, 6–12), with a higher risk of brain metastases occurring in the settings of adenocarcinoma and large-cell carcinoma (non-squamous histologies) (4–6, 13) as well as stage IIIB (as compared to stage IIIA) (9).

Brain metastases can be devastating to the patient, leading to impaired quality of life (QoL), worsened neurocognitive function (NCF), potential life-threatening conditions, and decreased survival (14). The response rate to whole-brain radiation therapy for brain metastases is only 50% (15, 16), with the survival of lung cancer patients with brain metastases remaining dismal, limited to a median of 3–18 months (4, 8, 15, 17, 18), despite advances in brain metastasis treatment.

The most commonly accepted means of reducing the incidence of brain metastases is the use of prophylactic cranial irradiation (PCI).

Prophylactic cranial irradiation is currently recommended for the management of small-cell lung cancer based on a large body of evidence. The use of PCI in limited-stage SCLC (LS-SCLC) was started in 1977 (19). Several randomized controlled trials (RCTs) (20–26) and an individual-patient-data-based meta-analysis (27) have shown PCI to significantly decrease the risk of brain metastasis [relative risk (RR) = 0.46] and improve overall survival (OS) (RR = 0.84) of SCLC patients in complete remission. While PCI, compared to no PCI, can cause neurotoxicity, it improves quality-adjusted life expectancy (28). Nowadays, in the absence of high quality evidence, PCI is typically started 4–6 weeks after completion of induction chemotherapy or chemoradiation in SCLC.

However, to date, PCI has not been shown to be associated with superior survival and is thus not routinely employed or recommended in the management of NSCLC. A Cochrane review published in 2005 and including four RCTs, compared PCI to no PCI in patients with NSCLC treated with a curative intent (29). This review showed that PCI significantly reduced the incidence of brain metastases in three of the four included RCTs, but none of the four included studies found an OS benefit. Additionally, no meta-analysis of the data was performed because of significant heterogeneity between the four trials included in the review, as stated by the authors (29–33).

### Objectives

This study aimed at systematically reviewing and, wherever possible, meta-analyzing the benefits and harms of PCI compared to no PCI in patients with NSCLC treated with a curative intent. It also aimed to explore whether the effects of PCI differed in the highest-risk NSCLC patients.

### Research Question

Is PCI beneficial in patients with NSCLC in terms of reducing the incidence of brain metastases, conferring an OS or disease-free survival (DFS) benefit, or improving QoL?

## Materials and Methods

### Study Design

We report this systematic review in accordance with the Preferred Reporting Items for Systematic Reviews and Meta-Analyses (PRISMA) guidelines (34).

### Participants, Interventions, Comparator

We included all prospective RCTs, with no restriction to language, publication date, or publication status (published, unpublished material, and abstracts).

We included trials recruiting participants with non-metastatic NSCLC of any age and stage who completed definitive locoregional therapy [a combination of surgery, and/or thoracic RT (dose > 30 Gy)] with or without chemotherapy, with complete response, partial response, or stable disease after therapy. We excluded studies including participants who were treated in a palliative intent.

We included studies that compared PCI to no PCI, irrespective of the PCI dose or RT technique used.

The outcomes of interest were incidence of brain metastasis, time-to-brain metastasis, OS, DFS, and QoL.

### Systematic Review Protocol

We developed a protocol that detailed the pre-specified objectives, eligibility criteria, outcomes of interest, search strategy, and analyses plan. We published the protocol in the International prospective register of systematic reviews (PROSPERO 2015: registration number CRD42015023982) (35).

### Search Strategy

We systematically searched the literature using the electronic databases EMBASE, MEDLINE, PubMed, and the Cochrane Central Register of Controlled Trials (CENTRAL). The search was run initially between 1946 and February 2014, then was updated until July 2016. We also received continual search alerts from PubMed through September 2017. We applied no limits for language. Search terms included “non-small-cell lung carcinoma,” “cranial irradiation,” and “randomized controlled trials.” The detailed search strategy can be found in Supplementary Data S1 in Supplementary Material.

We also searched the Cochrane Database for Systematic Reviews, and the following clinical trials registers: http://ClinicalTrials.gov, EU Clinical Trials Register (EU-CTR), the International Clinical Trial registry Platform, and the International Standard Randomised Controlled Trial Number registry, and contacted their principal investigators. Also, we screened the reference lists of relevant studies (trials or reviews) and pertinent books, as well as proceedings of oncology meetings [e.g., the American Society of Clinical Oncology (ASCO)]. Experts in the field were consulted for information on potential unpublished data.

### Data Sources, Studies Sections, Study Selection

After removing duplicate publications, a team of two reviewers screened the title and abstracts of all identified citations in duplicate and independently. We obtained the full text of citations judged as potentially eligible by at least one of the two reviewers. We screened all full texts for eligibility by teams of two reviewers in duplicate and independently. We used a pilot tested screening form. Disagreement between reviewers was resolved by consensus or by seeking the opinion of an expert in the field. When multiple reports for the same RCT were found, we retained the one with the most recent results. When different manuscripts or abstracts reported different outcomes for the same study, we included results from these different reports.

Two reviewers (Karine A. Al Feghali and Rami A. Ballout) conducted data extraction in duplicate and independently. They resolved disagreements by discussion. When agreement was not reached, the senior author (Fady B. Geara) made the final decision. Whenever needed, we attempted to contact trial authors to confirm accuracy of information, request missing information, or request information needed for subgroup analysis. When author contact was unsuccessful, we attempted to extract data from figures (e.g., Kaplan–Meier curves).

We used a standardized and pilot tested data abstraction form. We extracted the following data from each of the included trials:

– study design, year of publication
– inclusion/exclusion criteria
– characteristics of trial participants: age, gender, performance status, stage, type of NSCLC with histological confirmation of the diagnosis (adenocarcinoma, large-cell carcinoma, and/or squamous cell carcinoma), type of treatment received (surgery and/or chemotherapy and/or thoracic RT)
– characteristics of the intervention: dose and fractionation of PCI versus no PCI
– outcomes assessed: occurrence of brain metastasis, time-to-brain metastasis, OS, DFS, QoL (using a validated score), and NCF.

### Risk of Bias Assessment

We used the Cochrane Collaboration’s tool for assessing risk of bias in the included trials. This tool covers six domains of bias: (1) selection bias, as assessed by random sequence generation and allocation concealment, (2) performance bias, as assessed by blinding of participants and personnel, (3) detection bias, as assessed by blinding of outcome assessment, (4) attrition bias, i.e., incomplete outcome data, (5) reporting bias due to selective outcome reporting, (6) other biases due to problems not covered elsewhere (36). We looked for selective reporting within studies by comparing the outcomes reported in the published report to the outcomes outlines in the protocol, if available, or in abstracts of presentations that preceded publication of the study.

Two independent reviewers assigned a judgment of high, low, or unclear risk of bias for each of these six domains, and then provided a summary assessment for the risk of bias for each study. We did not exclude studies based solely on the risk of bias.

### Data Analysis

#### Summary Measures

We used relative risks (RR) for brain metastasis and the hazard ratios (HR) for OS and DFS. We derived HR based on Parmar et al., Spruance et al., and Guyot et al. methods (37–39). Supplementary Data S2 in Supplementary Material provides detailed information on the methods used to derive HR.

#### Meta-Analysis

We computed the pooled RR or HR and 95% confidence interval (CI) for each outcome using the random-effects model. We assessed statistical heterogeneity among trials using the Chi-squared tests with significance at *p*-value ≤ 0.1. We quantitatively assessed it using *I*^2^, which measures the degree of inconsistency across studies in a meta-analysis.

We interpreted the degree of heterogeneity accordingly to the value of *I*^2^ as follows: “low” for *I*^2^ below 25%, “moderate” for *I*^2^ below 50%, and “high” for *I*^2^ above 50%, respectively (40). We used the funnel-plot method to assess and correct for publication bias.

#### Additional Analyses

We planned to perform a subgroup analysis to evaluate whether the effects of PCI differs for the highest-risk patients (stage IIIA and IIIB), for the different radiation doses, and for the types of NSCLC histology (squamous versus non-squamous). However, we were not able to perform these analyses due to the lack of data.

We performed sensitivity analyses, excluding older studies (published before 1995), as we expected the potential benefit of PCI to be more evident in more recent studies after the introduction of cisplatin-based chemotherapy, and these latter studies to have a more rigorous methodology.

One of the eligible trials (by Umsawasdi et al.) did not provide the needed data for inclusion in the meta-analysis, so we derived HR by working backwards from the KM graph (using WebPlotDigitizer). We opted to exclude this study from the primary analysis and include it in a sensitivity analysis.

### Quality of Evidence Assessment

We assessed the quality of evidence (i.e., certainty of evidence) for the outcomes of interest using the Grading of Recommendations, Assessment, Development, and Evaluation approach (41). This instrument allows us to determine the extent to which one can be confident that an effect estimate truly represents reality. It depicts five factors that can lead to rating down the quality of evidence and three factors that can lead to rating up the quality of the evidence, from a starting point determined by study design. High likelihood of bias (42), inconsistency (43), indirectness of evidence (44), imprecision (45), and presence of publication bias (46) can all lead to downgrading of evidence. Factors that may lead to upgrading the quality of the body of evidence include a large magnitude of effect, a dose–response relationship, and plausible confounding that would reduce a demonstrated effect or suggest a spurious effect when results show no effect (47). Based on this appraisal, the body of evidence can be classified into four levels of quality (high, moderate, low, or very low) for each outcome of interest (41, 48).

## Results

### Flow Diagram

**Figure 1:**
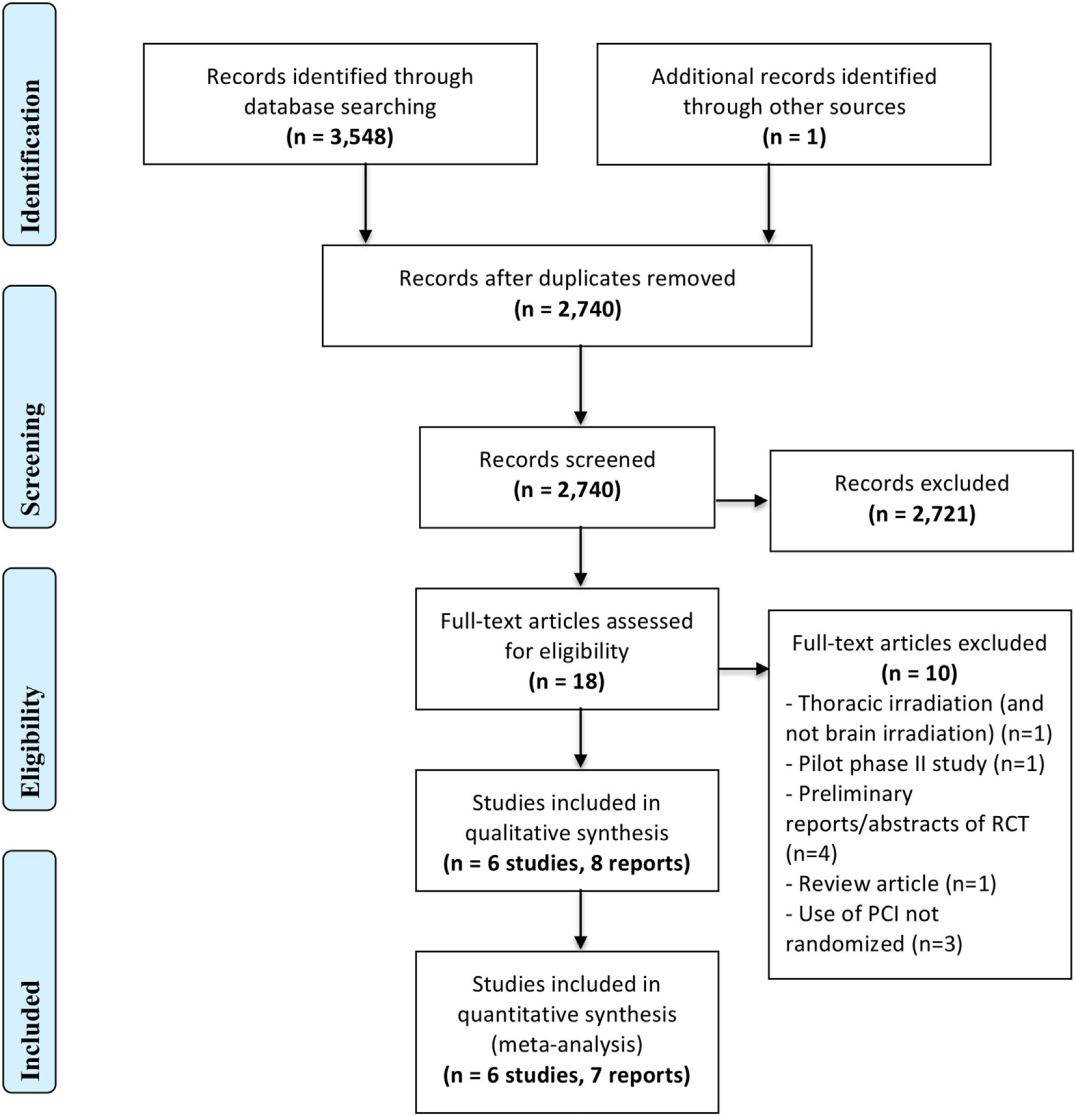
PRISMA Flow Diagram.

Figure 1 represents the PRISMA flow diagram.

### Study Selection and Characteristics

Our search strategy identified a total of 3,548 records, of which 2,741 remained after removal of duplicate records. Out of these records, we judged eight reports of six trials (30–33, 49–52) as eligible and included them in the qualitative analysis, with only seven of them (30–33, 49, 50, 52) entering into the quantitative analysis. One of the eight references is an updated report, in abstract form (50), of an earlier study. The seven reports included in the quantitative analysis had data on the incidence of brain metastasis as well as survival outcomes. Among the eight reports identified, only two reported QoL measures.

We excluded a total of 10 records at the full text screening stage for the following reasons: different fractionation for thoracic irradiation (and not brain irradiation) (*n* = 1), pilot phase II study (*n* = 1), preliminary reports/abstracts of RCT (*n* = 4, only final and updated reports, even if in abstract form, were included in the meta-analysis), review article (*n* = 1), and use of PCI not randomized (*n* = 3). We excluded Pottgen et al. (53) because the administration of PCI was not randomly allocated. Instead, randomization was performed on two curative treatment options, and patients in one of the two arms all received PCI.

Table 1 details the design, characteristics of participants (with inclusion and exclusion criteria), the treatment modality used, intervention and control arm details, outcomes assessed, and funding and conflicts of interest wherever reported, for the six included RCTs.

**Table 1:** Characteristics of included studies.

Reference	Study design and follow-up	Participants (including performance status and NSCLC stage ± histology)	Cure-intended treatment used	PCI technique and dose used (i.e., intervention details)	Control arm	Outcomes assessed (with outcome measures)	Funding and authors’ conflicts of interest
Cox et al. (30)	Randomized 4-arm randomized controlled trial (RCT) Clinical assessment and CXR monthly for the first 6 months after treatment, every 2 months for the next 18 months, and every 3 months thereafter Radionuclide brain scan if/when neurological symptoms develop	410 patients of any age with locally advanced *NSCLC and SCLC* with a KPS > 50 (data analysis was based on 323/410, 42/323 patients had SCLC and were excluded from this review) 40% squamous cell carcinoma, 10% adenocarcinoma, 17% large-cell carcinoma, and 20% other NSCLC histologies (13% with SCLC, excluded from this review)	Arm 1: intermediate-course chest RT (50Gy/25F/5 weeks) Arm 2: intermediate-course chest RT (50Gy/25F/5 weeks) + PCI Arm 3: short-course chest RT (42Gy/15F/3 weeks) Arm 4: short-course chest RT (42Gy/15F/3 weeks) + PCI *No chemotherapy was used in any arm*	PCI (arms 2 and 4) given as 20 Gy/10F/2 weeks	Cure-intended treatment without PCI (arms 1 and 3)	Incidence of brain metastases Time-to-brain metastases Median OS No formal assessment of QoL/toxicity was performed	Supported in part by the Veterans Administration and grant CA 23415-02 awarded by the National cancer Institute, and by an Interagency Agreement between the Veterans Administration and the National cancer Institute. No. Y01-CM-70107 COI: not reported
Umsawasdi et al. (31)	Randomized controlled 2-arm trial Radionuclide or CT scan for the brain if/when neurological symptoms develop Follow-up intervals unclear	100 patients with locally advanced non-small cell lung cancer 13% stage I/II 87% stage III 36% squamous cell carcinoma and 48% adenocarcinoma	Combined chemoradiotherapy as single curative treatment for active disease, or as an adjuvant therapy Thoracic RT use was not described	PCI (30 Gy/10F/2 weeks)	Cure-intended treatment without PCI	Incidence of brain metastases Time-to-brain metastases OS No formal assessment of QoL was performed	Supported in part by Grant CA 05831 Project 9A from the National Cancer Institute, NIH, USPHS, DHHS, Bethesda, Maryland and by Bristol Laboratories, Syracuse, New York COI: not reported
Russel et al. (32)	Randomized controlled 2-arm trial Clinical assessment every 3 months for all patients. CT brain if/when neurological symptoms develop, and in all patients surviving to 7.5 months following PCI completion regardless of presence/absence of neurological symptoms	200 patients with adenocarcinoma (67%) or large-cell carcinoma (33%) of the lung clinically confined to the chest (187 patients were evaluable)	161/187 received primary thoracic RT (55–60 Gy/30F/6 weeks) with no concurrent chemotherapy, while the remaining received postoperative RT (50 Gy/25F/5 weeks) after gross intrathoracic disease resection	PCI given concurrently with the sixth fraction of chest irradiation (30 Gy/10F/2 weeks)	Cure-intended treatment without PCI	Incidence of brain metastases Median, 1-year and 2-year OS	Funding: not reported COI: not reported
Miller et al. (33)	Randomized (2 × 2) 4-arm factorial RCT Monthly follow-up for all patients for the first year No data provided on other investigations performed	254 patients with unesectable *stage III* NSCLC 52% squamous cell carcinoma, 31% adenocarcinoma, and 17% large-cell carcinoma	Arm 1: chest RT alone (58 Gy/29F/6 weeks) Arm 2: chest RT + PCI Arm 3: chest RT + CT Arm 4: chest RT + CT + PCI	PCI (arms 2 and 4) given as 37.5 Gy/15F/3 weeks for the first 34 patients enrolled, and as 30 Gy/15F/3 weeks for the rest	Cure-intended treatment without PCI (arms 1 and 3)	Incidence of brain metastases Median OS No formal assessment of QoL was performed	Supported in part by PHS Cooperative Agreement grants awarded by the National Cancer Institute, DHHS COI: not reported
Gore et al. (49) [updated analysis in an abstract form in 2012 (50)]Sun et al. (51) (NCF and QoL analysis)	Randomized controlled 2-arm trial NCF was evaluated at baseline, 3, 6, 12, 18, 24, 30, 36, and 48 months after enrollment and then yearly afterward QoL was assessed at baseline, and coupled with brain imaging (CT or MRI) at 6, 12, 24, 36, and 48 months after enrollment and then yearly afterward Clinical assessment to all patients every 6 months for 2 years from the start of PCI, and then yearly afterward	356 patients aged 39–84 years old with *Stage III* (54% IIIA and 46% IIIB) NSCLC, of which 96% had an ECOG PS of 0–1 32% squamous cell carcinoma, 33% adenocarcinoma, 6% large-cell carcinoma, and 29% other NSCLC histologies	High-dose chest irradiation (RT; >30 Gy) with or without adjuvant or neoadjuvant, chemotherapy and/or surgical resection wherever applicable. RT with or without chemotherapy could be given pre- or post- operatively in surgical candidates	PCI (30 Gy/15F/3 weeks)	Cure-intended treatment without PCI	1-year and 5-year OS rates 1-year and 5-year DFS rates 1-year and 5-year incidences of brain metastases NCF and QoL	Funding: not reported COI: one author indicated financial interest in the study
Li et al. (52)	Randomized controlled 2-arm trial MRI of the brain and QoL assessment for all patients were done at baseline, followed by clinical assessment every 3 months for the first 2 years, and every 6 months thereafter Chest and upper abdomen CT, as well as brain MRI were performed every 6 months from enrollment to check for tumor relapse and/or metastasis, and were repeated whenever clinically indicated	156 patients with Stage IIIA-N2 NSCLC, of which 98% had an ECOG PS of 0–1 Median age was 55 (31–73) in the PCI arm versus 57 (24–75) in the control arm 25% squamous cell carcinoma, 62% adenocarcinoma, and 13% other NSCLC histologies	Surgical resection followed by adjuvant platinum-based chemotherapy	PCI (30 Gy/15F/3 weeks)	Cure-intended treatment without PCI	Incidence of brain metastases Median DFS Median OS QoL and toxicities	Supported by Guangdong Province Science and Technology project management (grant numbers 2005B3030 1002, 2010B031600064) COI: not reported

*NSCLC, non-small-cell lung cancer; PCI, prophylactic cranial irradiation; CT, computed tomography; RT, radiation therapy; Gy, Gray; F, fractions; OS, overall survival; QoL, quality of life; COI, conflict of interest; NCF, neurocognitive function; DFS, disease-free survival; SCLC, small cell lung cancer*.

A total of 1,373 patients with NSCLC were included in the meta-analysis. Most of these patients were males (62–77%), and most had a Karnofsky Performance Status of more than 70% or an Eastern Cooperative Oncology Group Performance Status (ECOG PS) of 0 or 1, indicating that most patients were ambulatory and able to care for self. Moreover, all of the included studies mandated histological or pathological confirmation in the diagnosis of NSCLC. Most of those patients had stage III of the disease; exclusively stage III in 3 of the included studies (33, 49, 52), 87% stage III in another study (31), and more than 70% stage III in a different one (32).

However, there were several important individual differences among the included trials. First, distribution of NSCLC histologies (adenocarcinoma versus squamous cell carcinoma, versus other histologies) differed markedly from one study to the other. For instance Li et al. included predominantly adenocarcinoma (52), while Miller et al. mostly included squamous cell carcinomas (33).

Second, curative treatment preceding PCI administration also differed between trials, with some that could be considered suboptimal based on today’s standards of care, such as old RT techniques, inadequate doses of thoracic RT, and suboptimal or no chemotherapy in a setting where it would have been otherwise indicated based on nowadays standards. For instance, the details of the curative therapy used in Umsawasdi et al., discussed in another publication (52), consisted of chemoradiation as a definitive treatment for 63 patients (thoracic RT to a dose of 50 Gy in 25 fractions), and a combination of surgical resection, chemotherapy, and thoracic RT for the other 34 patients. On the other hand, the patients in Cox et al. either received primary “short-course” lung RT (42 Gy in 15 fractions), or “intermediate-course” lung RT (50 Gy in 25 fractions), with no chemotherapy at all (30). Moreover, none of the patients in Miller et al. underwent surgery (33). They were either treated with thoracic RT alone (58 Gy in 29 fractions) or with neoadjuvant chemotherapy, followed by thoracic RT and adjuvant chemotherapy. RTOG 0214 (49) allowed all potentially curative therapy, defined as high-dose thoracic RT (>30 Gy) or surgery. Neoadjuvant, adjuvant, or concurrent chemotherapy was permitted, as well as pre- or postoperative RT. Finally, all patients in the study by Li et al. had complete resection (pneumonectomy in 15%, lobectomy in 84%, and bilobectomy in 1% of patients) followed by adjuvant chemotherapy (52).

Third, most studies mandated a radionuclide/radioisotopic brain scan (30, 31), a CT scan of the brain (30–32), or an MRI of the brain (49, 52) after completion of curative treatment and prior to study entry. However, Miller et al. did not mention any pretreatment brain imaging performed (33).

Most studies used 30 Gy (in 10 or 15 fractions) as a total dose for PCI, except the first 34 patients in Miller et al. who received 37.5 Gy in 15 fractions (33), and the patients in Cox et al. who were treated with 20 Gy in 10 fractions (30). The control arm in the six included studies consisted of observation after a potentially curative treatment.

The outcomes studied were the incidence of brain metastases and OS in all of the six included studies (30, 31, 33, 49, 52), DFS in two of the studies (49, 52), time-to-brain metastasis in three of the studies (30–32), QoL in two of the studies (51, 52), and NCF in one study only (51).

Heterogeneity between studies was also seen at the level of differences in follow-up protocols. In some studies, their protocols required brain imaging only if symptoms developed (30–32), while in the others, brain imaging was ordered routinely every 6 months regardless of symptom development (49, 52). As for Miller et al., it did not detail the investigations to be performed at the monthly follow-up visits following treatment completion (33).

### Risk of Bias Assessment

**Figure 2:**
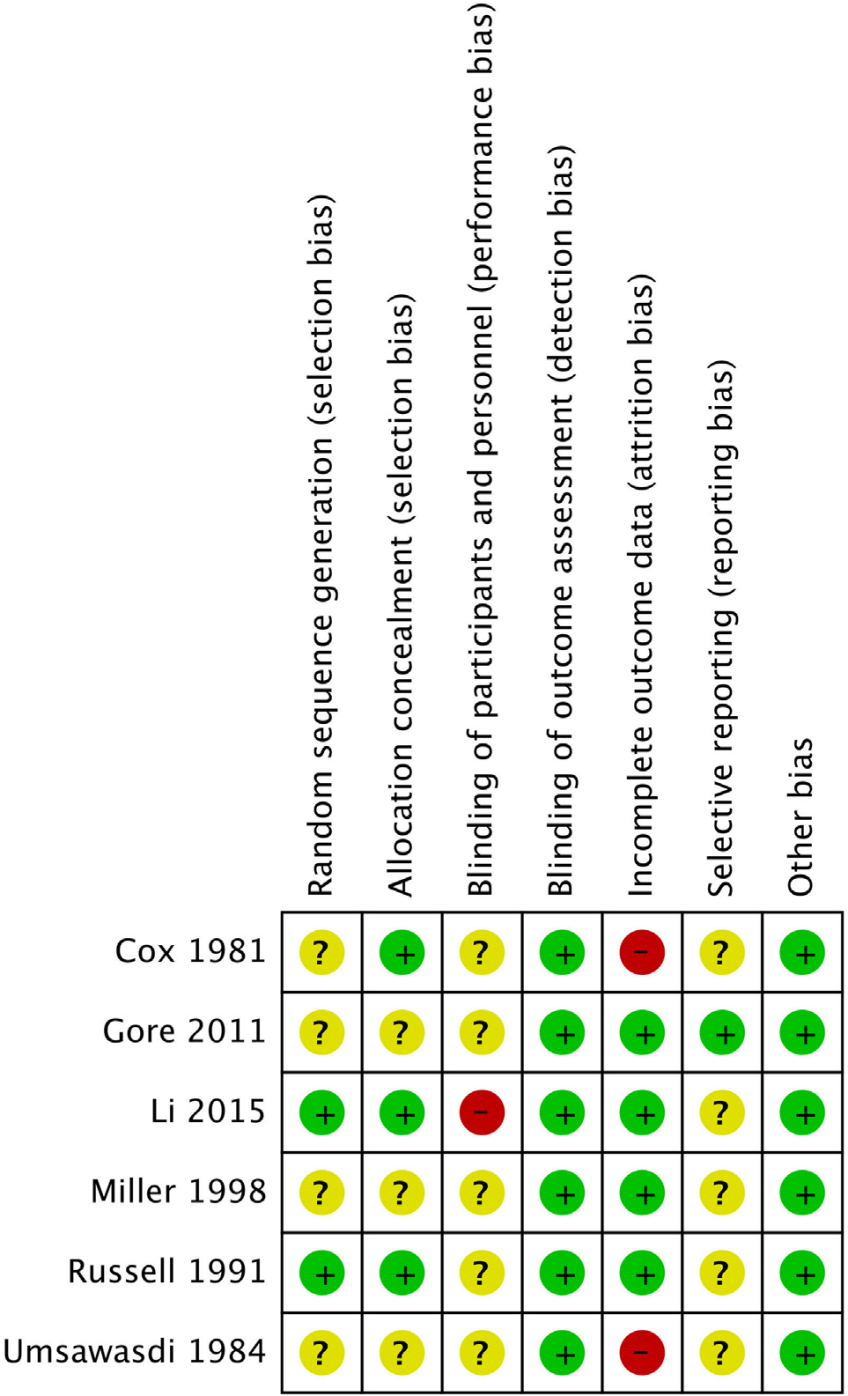
Risk of bias assessment for the included studies.

Risk of bias assessment across all studies is shown in Figure 2 and detailed in Table 2. Of the six trials included in our review, only two had adequate random sequence generation (32, 50), while the remaining four had an unclear randomization method (30, 31, 33, 49). Moreover, allocation concealment was adequate in only three of the included studies (30, 32, 52), and inadequate in the other three (30, 31, 33, 49). However, the blinding of participants and personnel was not feasible for testing such an invasive procedure, and thus, all studies were “open-label.” Similarly, blinding of outcome assessment was not performed in all of the included studies, but detection bias is not a concern in here since the outcomes of brain metastases and mortality are objective binary outcomes.

**Table 2:** Risk of bias assessment for the included studies.

Reference	Random sequence generation (selection bias)	Allocation concealment (selection bias)	Blinding of participants and personnel (performance bias)	Blinding of outcome assessment (detection bias)	Incomplete outcome data (attrition bias)Use of an intention-to-treat (ITT) analysis	Selective outcome reporting (reporting bias)	Overall risk of bias
Cox et al. (30)	*Low to unclear risk“ *Patients were centrally randomized by telephone call,” though precise randomization method used was not specified	*Low risk* Central randomization of patients was done through telephone calls at the Statistical center of the Frontier Science and Technology Research Foundation	*Unclear risk* No data reported	*Low risk* Although there were no data reported on this, the outcomes assessed were objective (most being binary in nature)	*High risk* 87/410 patients enrolled in the study were excluded from the analysis Despite reasons of exclusion being clearly stated and justified, imbalances in baseline characteristics may have resulted across the study arms due to the large number of excluded patients. The study also did not employ an ITT analysis	*Unclear risk* No published protocol	*Unclear risk*
Umsawasdi et al. (31)	*Unclear risk* Randomization method not specified, despite the study’s title “prospective randomized study”	*Unclear risk* No data reported	*Unclear risk* No data reported	*Low risk* Although there were no data reported on this, the outcomes assessed were objective (most being binary in nature)	*High risk* 3/49 patients randomized to receive PCI did not receive it and were excluded from the analysis, with one of them developing brain metastasis during treatment This indicates that the study did not employ an ITT analysis, with likely presence of attrition bias due to the exclusion of the patient developing one of the study’s primary outcomes	*Unclear risk* No published protocol	*Unclear risk*
Russel et al. (32)	*Low risk* “The randomization scheme described by Zelen was used to achieve institutional balance and incorporated three patient-related stratifications: prior surgery, pretreatment KPS, and histology”	*Low risk* “Following registration and confirmation of eligibility, patients were randomly assigned by RTOG headquarters”	*Unclear risk* No data reported	*Low risk* Although there were no data reported on this, the outcomes assessed were objective (most being binary in nature)	*Low risk* Data reported and analyzed for all 187 patients enrolled in the study and the study stated it employed ITT analysis “All the analyses were based upon the intention-to-treat principle”	*Unclear risk* No published protocol	*Low risk*
Miller et al. (33)	*Unclear risk* Randomization method not specified	*Unclear risk* Given that patients were stratified based on performance status and/or histology prior to assignment of treatment, it is possible that those with lower performance status or worse histology were excluded from intensive treatment arms	*Unclear risk* No data reported	*Low risk* Although there were no data reported on this, the outcomes assessed were objective (most being binary in nature)	*Low risk* 28/254 patients enrolled in the study were excluded from the analysis being declared as ineligible, with reasons of exclusion being clearly stated and justifiedThis study seems to employ ITT analysis, though not explicitly stated	*Unclear risk* No published protocol	*Unclear risk*
Gore et al. (49) [updated analysis in an abstract form in 2012 (50)]Sun et al. (51) (NCF and QoL analysis)	*Unclear risk* “Randomly assigned to either PCI or observation,” though precise randomization method used was not specified	*Unclear risk* Given that patients were stratified based on stage (IIIA versus IIIB), histology (non-squamous versus squamous), and/or type of therapy (surgery or not) prior to assignment of treatment, it is possible that those with poorer overall prognosis were excluded from intensive treatment arms	*Unclear risk* No data reported	*Low risk* Although there were no data reported on this, the outcomes assessed were objective (most being binary in nature)	*Low risk* Only 3/340 of the patients enrolled in the study were not followed-up after they withdrew consent, with data being reported for all the rest (i.e., nearly complete outcome data) with the study employing an ITT analysis as illustrated in its CONSORT diagram	*Low risk* The study reported data on all outcomes pre-set in its published protocol on http://clinicaltrials.gov (NCT00048997)	*Low risk*
Li et al. (52)	*Low risk* The study used a minimization procedure with stratification by histology (squamous versus non-squamous), center, and ECOG PS (0–1 versus 2)	*Low risk* “Random assignment instructions were obtained through an independent provider by telephone”	*High risk* Given that the study was an open-label trial, neither the participants nor the personnel were blinded	*Low risk* Although there were no data reported on this, the outcomes assessed were objective (most being binary in nature)	*Low risk* Data were reported and analyzed for all 156 patients enrolled in the study, and the study employed an ITT analysis as illustrated in its CONSORT diagram	*Unclear risk* No published protocol	*Low risk*

*PCI, prophylactic cranial irradiation; KPS, Karnofsky Performance Status; RTOG, radiation therapy oncology group; CONSORT, consolidated standards of reporting trials; ECOG PS, Eastern cooperative oncology group performance status; NCF, neurocognitive function*.

Incomplete patient data were improperly addressed in two of the included studies (30, 31). In Umsawasdi et al., three patients were excluded from the intervention group in the trial, and one of these three patients developed brain metastasis. In Cox et al., a significant number of patients refused PCI, and the loss of these patients might have been related to the trial’s outcome measures, thus possibly introducing attrition bias.

Intention-to-treat analysis was performed in four of the trials (32, 33, 49, 50), and incomplete outcome data were adequately assessed in these trials.

Furthermore, only one study (RTOG 0214) (49) had predefined outcomes specified in a published protocol on www.rtog.com and http://clinicaltrials.gov, thus obviating the risk of reporting bias. The remaining five studies were all at high risk for reporting bias as no protocols were found for them.

### Synthesized *Findings*

#### Incidence of *Brain Metastasis*

All of the six included studies individually showed a reduction in the incidence of brain metastases with PCI as compared to no PCI, with most being significant (30, 31, 33, 50, 52), and one not significant (32) (Table 3).

**Table 3:** Summary of results—incidence of brain metastases and survival—extracted from the trials on NSCLC included in this systematic review and meta-analysis (PCI versus no PCI).

					Brain metastases (%)			Median survival (months)/overall survival % at [X year(s)]		
	
Reference	Primary (cure-intended) therapy	Stage	PCI dose	*N*	PCI	Control	*p*-Value	PCI	Control	*p*-Value
Cox et al. (30)	RT only	Inoperable	20 (2 Gy × 10)	281	7/136 (6%)	16/145 (13%)	0.038	8.2 months	9.7 months	0.5
Umsawasdi et al. (31)	Triple modality (surgery + RT + CT)	I–II (13%) III (87%)	30 (3 Gy × 10)	97	2/46 (4%)	14/51 (27%)	0.002	22% (3 years)	23.5% (3 years)	Not reported
Russell et al. (32)	RT only	I/III	30 (3 Gy × 10)	187	8/93 (9%)	18/94 (19%)	0.1	8.4 months 40% (1 year) 13% (2 years)	8.1 months 44% (1 year) 21% (2 years)	0.36
Miller et al. (33)	RT + CT	III	30 (2 Gy × 15) 37.5 (2.5 Gy × 15)	226	1/111 (1%)	13/115 (11%)	0.003	8 months	11 months	0.004
Gore et al. (49) [2012 abstract (50)]	Triple modality (surgery + RT + CT)	IIIA (54%) IIIB (46%)	30 (2 Gy × 15)	340	19/163 (17.3%)	39/177 (26.8%)	0.009	75.6% (1 year) 26.1% (5 years)	76.9% (1 year) 24.6% (5 years)	0.57
Li et al. (52)	Surgery + CT	IIIA-N2	30 (3 Gy × 10)	156	10/81 (12%)	29/75 (39%)	<0.001	31.2 months 44.5% (3 years) 27.4% (5 years)	27.4 months 38.7% (3 years) 22.8% (5 years)	0.310

*NSCLC, non-small cell lung cancer; PCI, prophylactic cranial irradiation; RT: radiation therapy; Gy: Gray; CT: chemotherapy*.

**Figure 3:**
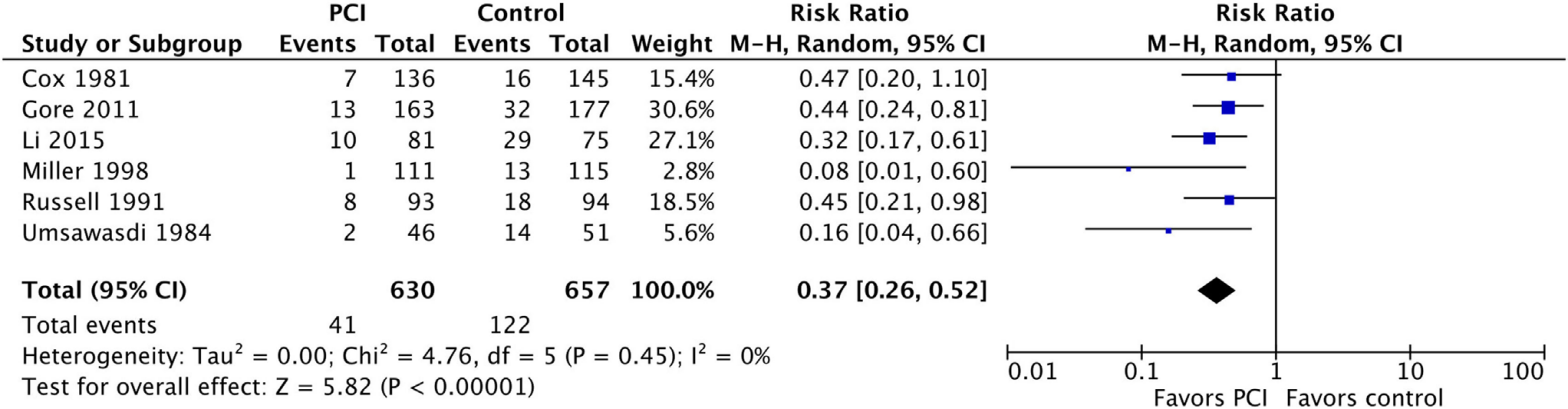
Effect of prophylactic cranial irradiation (PCI) on the incidence of brain metastases in 1,287 patients with non-small-cell lung cancer from six trials. Abbreviations: RR: relative risk; CI: confidence interval.

**Figure 4:**
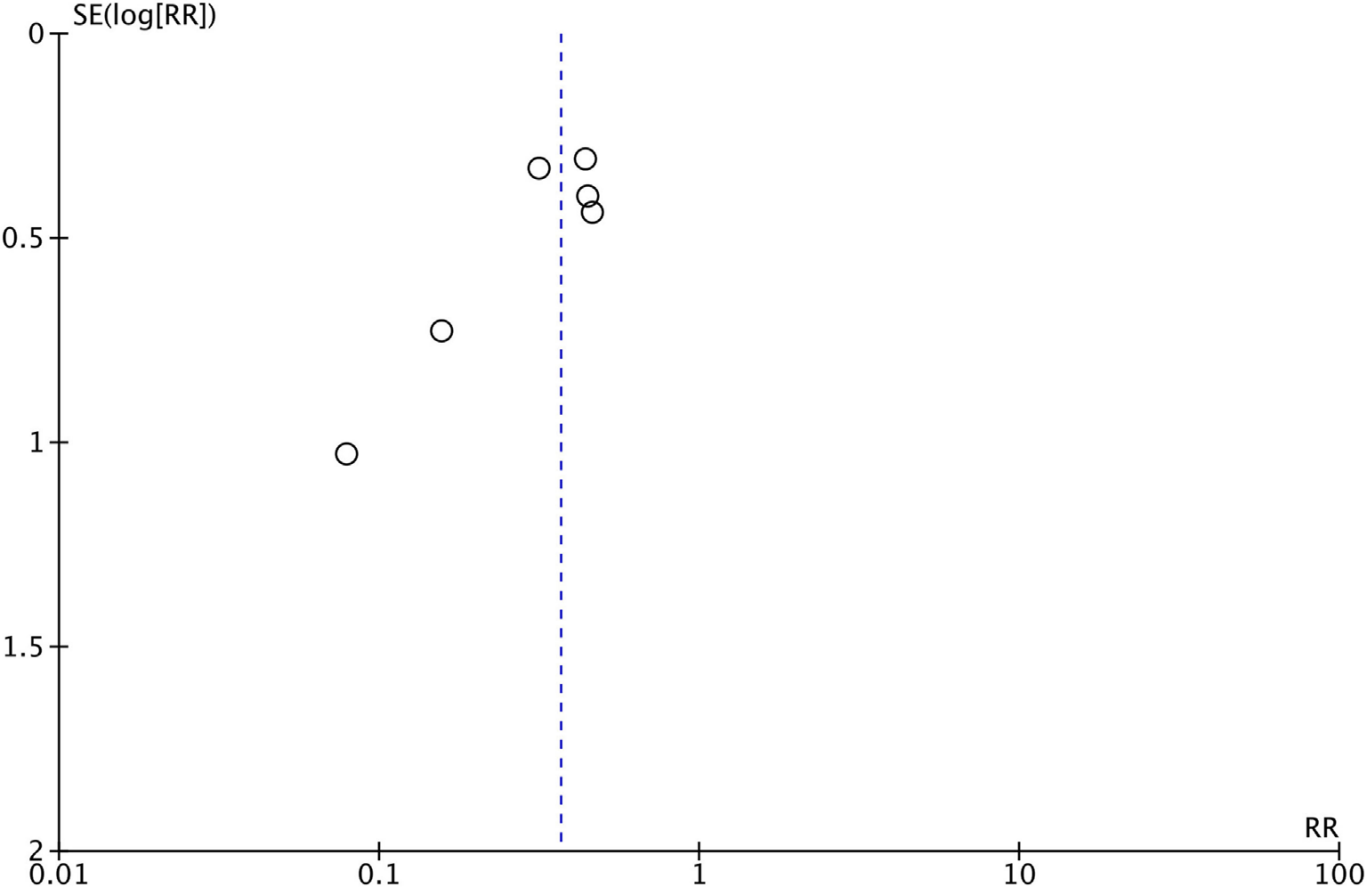
Inverted funnel plot for trials addressing the incidence of brain metastases. Abbreviation: RR, relative risk.

The meta-analysis for this outcome included data from six studies (Figure 3) with a total of 630 patients in the PCI arm and 657 patients in the no PCI arm. PCI was associated with a significant reduction in the rates of brain metastases as compared to no PCI [Relative risk (RR) = 0.37; 95% CI: 0.26–0.52; *I*^2^ = 0%]. The inverted funnel plot did not suggest publication bias (Figure 4). We rated the quality of evidence as moderate quality due to the high risk of bias (Table 4).

**Table 4:** Assessment of the quality of the evidence for each outcome using GRADE.

Quality assessment							No. patients		Effect		Quality	Importance
No. studies	Study design	Risk of bias	Inconsistency	Indirectness	Imprecision	Other considerations	PCI	Control	Relative (95% CI)	Absolute (95% CI)		
**Incidence of brain metastases**												

6	RCTs	Serious^a^	Not serious	Not serious	Not serious	–	630	657	RR 0.31 (0.20, 0.46)	128 fewer deaths per 1,000 (from 100 fewer to 148 fewer)	Moderate	Critical

**OS**												

5	RCTs	Serious^a^	Not serious^b^	Not serious	Serious^c^	–	630	657	HR 1.08 (0.90, 1.31)	70 more deaths per 1,000 (from 87 fewer to 270 more)	Low	Critical

**DFS**												

2	RCTs	Not serious	Not serious	Not serious	Not serious	–	252	244	HR 0.78 (0.64, 0.96)	174 fewer deaths per 1,000 (from 32 fewer to 284 fewer)	High	Critical

**QoL/NCF**												

2	RCTs	Not serious	Not serious	Not serious	Not serious	Different QoL and NCF instruments were used in the two studies, thus results could not be combined in a meta-analysis	252	244	QoL—no differences in QoL deterioration between PCI and no PCI arms NCF—only difference in NCF analysis was in the HVLT with greater deterioration in immediate recall (*p* = 0.03) and delayed recall (*p* = 0.008) at 1 year with PCI		High	Important

GRADE, Grading of Evidence, Assessment, Development and Evaluation; CI, confidence interval; RCT, randomized controlled trial; RR, relative risk; HR, hazard ratio; RCT, randomized controlled trial; OS: Overall survival; DFS: Disease-free survival; QoL: Quality of life; NCF: Neurocognitive function; HVLT: Hopkins Verbal Learning Test

*^a^Three out of the six studies included had low risk of bias; the other three studies had unclear risk of bias*.

*^b^I2 was 46% indicating moderate level of heterogeneity. This was taken into account along with the borderline risk of bias by downgrading the level of evidence by one level. This downgrading has been applied to the risk of bias criteria*.

*^c^The CI includes values that indicate benefit, and others that indicate harm*.

#### Overall *Survival*

**Figure 5:**
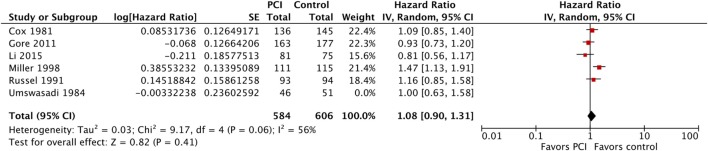
Effect of PCI on overall survival in 1,190 patients with non-small-cell lung cancer from five trials. Abbreviations: PCI, prophylactic cranial irradiation; CI, confidence interval.

**Figure 6:**
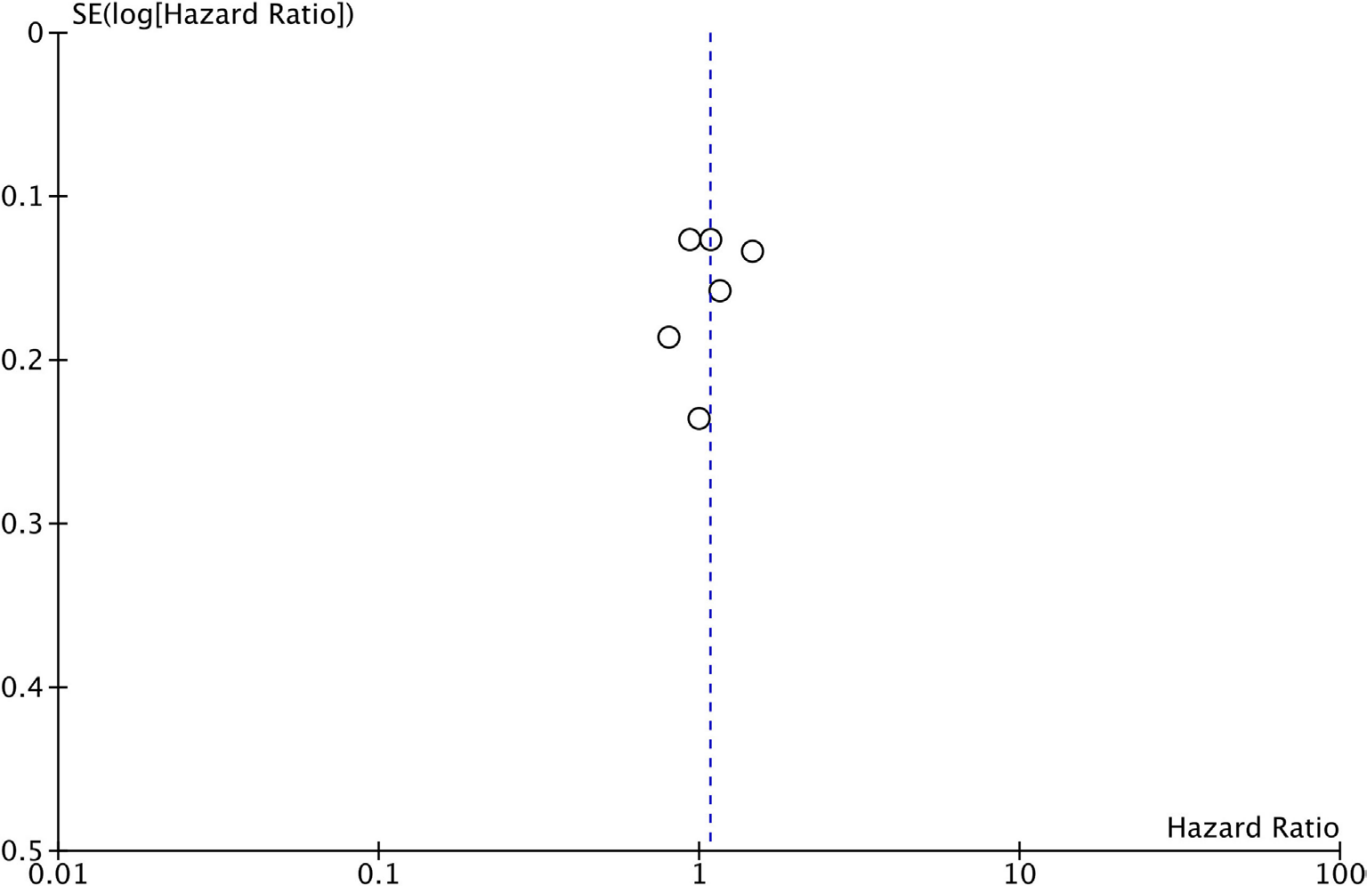
Inverted funnel plot for trials addressing overall survival.

Five of the six included studies contributed data to the meta-analysis for OS (Figure 5; Table 3), with a total of 584 patients in the PCI arm and 606 patients in the no PCI arm. The pooled HR was 1.08 (95% CI: 0.90–1.31; *I*^2^ = 56%). Figure 6 shows the inverted funnel, which does not suggest publication bias.

**Figure 7:**
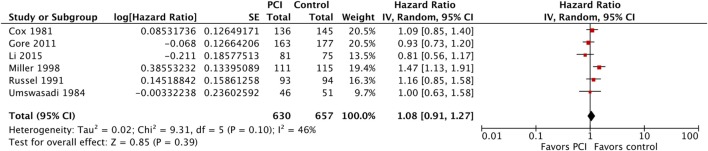
Sensitivity analysis: Effect of PCI on overall survival in 1,287 patients with non-small-cell lung cancer from six trials (Umsawasdi et al. added). Abbreviations: PCI, prophylactic cranial irradiation; CI, confidence interval.

**Figure 8:**
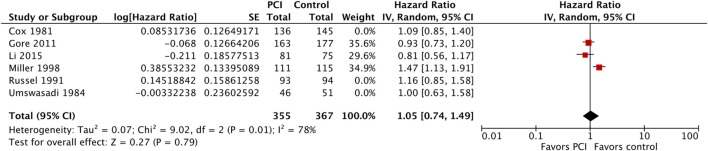
Sensitivity analysis: effect of PCI on overall survival in 722 patients with stage III non-small-cell lung cancer from three trials published after 1995. Abbreviations: PCI, prophylactic cranial irradiation; CI, confidence interval.

Moreover, we performed a sensitivity analysis where we included Umsawasdi et al. (31), which was excluded from the primary analysis as its HR was derived working backwards form the Kaplan–Meier graph (Figure 7). The point estimate and CI were very close to the ones from the primary analysis (HR = 1.08, 95% CI: 0.91–1.27; *I*^2^ = 46%). We also conducted another sensitivity analysis restricted to studies published after 1995 (44, 46, 50), when platinum-based chemotherapy added to RT became the standard of care for locoregionally advanced NSCLC (54–58) (Figure 8). The three studies included in this sensitivity analysis included patients with stage III NSCLC exclusively. The sensitivity meta-analysis showed no significant difference in OS between PCI and no PCI groups (HR = 1.05, 95% CI: 0.74–1.49; *I*^2^ = 78%). We rated the quality of evidence as low due to high risk of bias, as well as imprecision of the data (Table 4).

#### Disease-*Free Survival*

**Figure 9:**
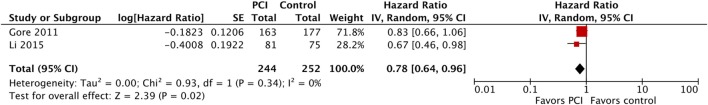
Effect of PCI on disease-free survival in 496 patients with stage III non-small-cell from two recent trials. Abbreviations: PCI, prophylactic cranial irradiation; CI, confidence interval.

The meta-analysis for DFS included data from two studies (three reports) (49, 52, 59) that included a total of 244 patients with stage III NSCLC in the PCI arm and 252 patients in the no PCI arm (Figure 9). Our meta-analysis demonstrated a significantly improved DFS with PCI compared to no PCI (HR, 0.78; 95% CI: 0.64–0.96; *I*^2^ = 0%). We rated the quality of evidence as high, since the evidence was direct, precise, consistent, and free of biases (Table 4).

#### Time-to-Brain Metastasis

While three studies reported time-to-brain metastases, we could not conduct a meta-analysis due to lack of adequate data from all studies (30–32). In Umsawasdi et al., the median time-to-brain metastasis was prolonged in the PCI group (50.5 weeks) compared to no PCI (23 weeks; *p*-value = 0.002) (31). Similarly, the median time for development of brain metastases in Cox et al. was also prolonged, but from 29 weeks in the no PCI group to 34 weeks in the PCI group (statistical significance not reported) (30). Moreover, PCI use was also stated to delay the onset of brain metastasis in Russel et al. but no further information on duration of delay was provided in their study (32).

#### Brain *Metastasis as First Site* of Recurrence–Relapse Pattern

Three of the included studies reported data on the patterns of failure/recurrence (31, 49, 52). However, we could not conduct a meta-analysis due to differences in the way this outcome was reported across studies. In Umsawasdi et al., the brain was the first site of relapse in 12 out of the 14 patients who developed brain metastasis in the control arm compared to none of the 2 patients who developed brain metastases in the PCI arm (31). Similarly, brain metastasis as a component of first failure occurred in 23% of patients not receiving PCI versus 10% of patients receiving PCI in Gore et al. (49). In the same study, brain metastasis as the only site of failure was reported in 21.5% in the control arm (no PCI) versus 9.1% in the intervention arm (PCI) (49, 50). Moreover, in Li et al., the crude 5-year brain relapse as first site of recurrence was 33.3% in the control arm (no PCI), as compared to 9.9% in the PCI arm (*p*-value < 0.001) (52).

#### Toxicities, QoL, and NCF

The acute toxicities due to PCI reported in the included studies were mainly epilation and acute skin reaction (32), grade 3 headaches (1%) (52), and fatigue (2%) (52).

However, most of the included trials reported late toxicities, with more or less details. We, however, were not able to conduct a meta-analysis as toxicities were not graded in all studies, and some studies only reported toxicities occurring in the PCI arm.

In Miller et al., there were “no excess neurological toxicities” in patients treated with PCI as compared to those in the no PCI arm (33), however, the definition of neurological toxicity was unclear. Similarly, Umsawasdi et al. reported that no late neurological complications were noted, although no formal neurologic assessment was conducted (31). In RTOG 0214 on the other hand, four patients in the PCI arm developed Grade 3 late toxicities (syncope, weakness, fatigue), without any late toxicities greater than Grade 3 (49). Similarly, in Li et al., the most commonly encountered late toxicities in the PCI arm were moderate headache or great lethargy (11.1%), severe headache (2.5%), grade 3 skin atrophy (one patient), and grade 3 fatigue (one patient) (52). In these last two studies, there was no mention about toxicities in the control arm. Finally, toxicities were not addressed in Cox et al. (30).

Late neurological complications due to PCI and QoL have only been formally addressed in two trials (51, 52). However, because these two studies used different QoL tools, it was not possible to combine them in a meta-analysis. Li et al. assessed QoL by means of the Functional Assessment of Cancer Therapy-Lung questionnaire for 129 out of 156 randomized patients (70 in the PCI arm, 59 in the control arm). No significant differences in QoL deterioration were found between the two groups (52). On the other hand, RTGO 0214 assessed QoL using the European Organisation for Research and Treatment of Cancer (EORTC) core tool (QoL Questionnaire-QLQC30) and brain module (QLQBN20) (51). This study too, found no statistically significant differences at 6 or 12 months in any component of the EORTC-QLQC30 or QLQBN20 scale (*p* > 0.05) as compared to baseline. However, the study notes “a trend” toward greater decline in patient-reported cognitive functioning in the PCI arm compared to no PCI (unadjusted *p* = 0.02 at 6 months, adjusted *p* = 0.24) (51). The same study (RTOG 0214), reported no significant difference in NCF deterioration, as determined by MMSE, between the two arms. Similarly, the percentage of patients who remained independent (as measured by the Activity of Daily Living Scale) at 12 months was not different between the two arms (*p* = 0.88). The only significant difference in the NCF analysis was in the Hopkins Verbal Learning Test (HVLT), whereby patients who received PCI showed a greater deterioration in immediate recall (*p* = 0.03) and delayed recall (*p* = 0.008) at 1 year compared to no PCI (51). We rated the quality of evidence as high, since the evidence was direct, precise, consistent, and free of biases (Table 4).

## Discussion

### Summary of Main Findings

This review shows that PCI, compared to no PCI, significantly decreases the incidence of brain metastases in NSCLC (by approximately 70%) and improves DFS in stage III patients. However, it appears to have no effect on OS or QoL, although it can result in some radiation-induced cognitive impairment.

Our finding that PCI significantly decreases the rates of brain metastases in patients with NSCLC compared with no PCI is in agreement with the findings of two previous systematic reviews (29, 60). While our meta-analysis found no significant difference in OS between the PCI and no PCI arms in NSCLC patients (HR = 1.08, 95% CI: 0.91–1.27), the meta-analysis by Xie et al. found a detrimental effect of PCI on OS with a HR of 1.19 (95% CI: 1.06–1.33, *p* = 0.004) (60). Our results likely differ from those reported by Xie et al. (60) because of differences in the included studies. Xie et al. (60) included a study by Pottgen et al. which is a RCT of primary resection followed by adjuvant thoracic RT versus preoperative chemotherapy followed by concurrent chemoradiotherapy, and not of the use of PCI (53). Our meta-analysis included the more contemporary study by Li et al. (52), did not include the study by Pottgen et al. (53) or any other non-randomized studies. Also, the forest plot in their manuscript shows the incorrect use of a HR of 1.07 for the trial by Gore et al., favoring no PCI (49, 50). The reviewers should have included the inverse of this ratio, because Gore et al. used the PCI group and not the control group as the reference level in their analysis.

A non-randomized, population-based study using the Surveillance, Epidemiology, and End Results database also addressed the effect on PCI on survival in NSCLC patients. It included a total of 17,852 patients with NSCLC, among whom only 1.8% received PCI as part of their treatment. No statistically significant difference in survival was found between the patients who received PCI and those who did not (HR, 1.04; 95% CI: 0.93–1.16), even in subgroups of patients at higher risk of brain metastases (patients younger than 60 years, adenocarcinoma histology, or stage IIIB) (61). We were unable to perform subgroup analysis in our meta-analysis because in most trials, results were not stratified by histology, stage, or response to induction chemotherapy (when applicable). Preliminary results from NVALT-11 study, which randomized 195 patients with radically treated stage III NSCLC to PCI or observation, were presented at the 2017 ASCO meeting (62), and at the 2017 International Association for the Study of Lung Cancer (IASLC) World Conference on Lung Cancer (WCLC) (63). This study also demonstrated a decrease in the incidence of symptomatic brain metastases (29.7 versus 8.1%), with no OS benefit (62), and no significant differences in Grade 3–4 toxicities (62). DFS was not reported in both abstracts. When the full manuscript of that RCT is published, we might be able to update our meta-analysis, if subgroup analyses are made available.

Our review found no deterioration in patients’ QoL with the use of PCI. In contrast, previous studies have demonstrated that whole-brain radiotherapy in patients with brain metastases was associated with neurocognitive deterioration which preceded QoL decline by 9–153 days (64). Although RTOG 0214 showed a decline in NCF based on HVLT-DR with PCI, there were no significant differences in QoL in that trial between the patients who received PCI and those who did not (59).

The multicenter trial by Pottgen et al. compared two different locoregional treatment strategies in patients with operable stage III NSCLC. In the first arm, patients underwent surgery followed by adjuvant thoracic RT. In the second arm, therapy consisted of induction chemotherapy, followed by concurrent chemoradiation and then surgery. All patients in the second arm received PCI (30 Gy in 10 fractions). However, patients randomized to the second arm received a more aggressive locoregional treatment (trimodality approach) than patients in the first arm. In that study, PCI successfully reduced the rate of brain metastasis as first site of failure, and the overall brain relapse rate. However, there was no significant difference in neurocognitive performance between the PCI and control arms (53). This is the only study, besides RTOG 0214 (included in our meta-analysis) (51) that reported on NCF in the setting of PCI for NSCLC. Another study by Gondi et al. was not included in this systematic review as it pooled QoL and NCF results from two RTOG randomized studies: RTOG 0214 (already included in this review), and RTOG 0212 (65), and randomized patients with limited-stage SCLC to standard-dose versus higher-dose PCI. PCI was associated with a higher risk of decline in self-reported cognitive functioning (SRCF) at 6 (OR 3.60, 95% CI: 2.34–6.37, *p* < 0.0001) and 12 months (OR 3.44, 95% CI: 1.84–6.44, *p* < 0.0001). PCI was also associated with a significant decline on HVLT-Recall and HVLT-Delayed Recall at 6 and 12 months, but was not closely correlated with decline in SRCF at the same time points (*p* = 0.05 and *p* = 0.86, respectively). PCI was not associated with a decline in global health status/QoL or any other EORTC QLQC30 symptom or functional scales. Age >60 years was associated with higher rates of HVLT-DR decline at 12 months (59). These results show that the PCI-induced cognitive decline is not only captured on formal memory testing like HVLT but is also, and more importantly, self-reported, and thus experienced by the patient.

The first systematic review published on the use of PCI in NSCLC was a Cochrane review (29). The two major limitations of that review are the absence of meta-analysis due to the heterogeneity of the trials included at that time, and the fact that it has not been updated since 2010 and, therefore, does not include the two most recent RCTs: Gore et al. (RTOG 0214) (49, 50) and Li et al. (52).

Brown et al. published another systematic review on the same topic in the form of two abstracts in 2015 (66, 67). However, the full text of that review has not been published. In that review, the search strategy also does not seem to be sensitive and exhaustive as it identified only 112 citations, compared to the 2,740 records that our search captured (Figure 1). Moreover, method-wise, they chose 1-year survival as one of their primary endpoints, thus limiting their data to a single survival estimate at one point in time, instead of taking the entire survival curve into consideration by using HRs as recommended by Parmar et al. (37).

Our study has a number of strengths. First, the systematic review methodology is rigorous and in accordance with the Cochrane Handbook of Systematic reviews, the search strategy is thorough and exhaustive, and the data included are up-to-date, covering the most recent trials conducted on the topic. Our study also fills a knowledge gap; it is the only meta-analysis done on stage III patients specifically, although formal subgrouping—stage III versus I–II and stage IIIB versus IIIA, pre-specified in the protocol, was not made possible by the available data. To elaborate, we believe that the therapeutic ratio of benefits versus risks is more advantageous in this group of patients (stage III) compared to those with earlier stages of NSCLC, because the propensity for brain metastases in the stage III patients is much greater. However, results of our meta-analysis showed otherwise; even in this specific subset of patients, PCI seems to have no OS benefit, despite a significant DFS benefit.

### Limitations

Our review is not free of limitations. First, we were unable to perform all the planned subgroup analyses because most corresponding authors of the included trials did not answer our e-mails. In particular, we were not able to stratify by stage (stage IIIA versus stage IIIB) or histology.

Moreover, in Cox et al. (30), 13% of the patients included had SCLC, and results were provided for NSCLC and SCLC separately for the endpoints of incidence of brain metastases and time-to-brain metastasis, but not for OS (30). Thus, this may have introduced an error in the reporting of OS, which has probably been diluted when combining all the trials together in the meta-analysis shown in Figure 6. Another limitation of our review is that, in the current targeted therapy and immunotherapy era, some of the drugs, such as erlotinib, gefitinib, and durvalumab can effectively cross the blood–brain barrier, and thus, reduce the incidence of brain metastases in NSCLC (68–75), thus potentially lessening the reported effect of PCI on the incidence of brain metastases and maybe dampening the DFS benefit.

Overall, in our review, PCI was definitely shown to change the failure pattern in NSCLC, from failing in the brain first to failing outside of the brain. However, more information is still needed from more RCTs to determine whether a specific subset of patients might derive a survival benefit from PCI versus no PCI compared to the rest of patients. Patients at high risk for brain metastases include (1): patients with superior sulcus tumors, also known as Pancoast tumors, who have a 40% risk of failing in the brain (76, 77), (2) patients with operable stage IIIA-N2 NSCLC, and/or patients with non-squamous histology (6, 78, 79), and (3) patients with a complete pathological response to neoadjuvant therapy (80). Other literature gaps include trials addressing the impact of PCI on QoL and NCF.

Supplementary Data S3 in Supplementary Material lists all the ongoing trials on this topic that are trying to address these gaps.

### Conclusion

In summary, there is moderate quality evidence that the use of PCI in NSCLC decreases the risk of brain metastases, but does not provide an OS benefit. However, data limited to stage III patients suggest that PCI improves DFS, with no effect on QoL. More evidence is still needed for us to be more confident about the benefits versus harms of PCI in NSCLC. It would be important to incorporate prospective NCF testing in all future studies. Efforts to identify high-risk groups, using predictive biomarkers might also help in the selective application of PCI.

## Author Contributions

KF designed and directed the project, and contributed to the implementation of the research, to acquisition of the data, analysis of the results, and writing of the manuscript. RB and AK contributed to the analysis of the results and to the writing of the manuscript. FG and EA contributed to the conception, design and implementation of the project, and revised the manuscript.

## Conflict of Interest Statement

The authors declare that the research was conducted in the absence of any commercial or financial relationships that could be construed as a potential conflict of interest.
