# Supplementary material for: Prophylactic Cranial Irradiation in Patients With Non-Small-Cell Lung Cancer: A Systematic Review and Meta-Analysis of Randomized Controlled Trials
Source: Front Oncol. 2018 Apr 20;8:115. doi: 10.3389/fonc.2018.00115 (PMC5919944; doi:10.3389/fonc.2018.00115)
Supplement: Supplementary file 1 [file Data_Sheet_1.docx]

Supplementary Material

PROPHYLACTIC CRANIAL IRRADIATION (PCI) IN PATIENTS WITH NON-SMALL-CELL LUNG CANCER (NSCLC): A SYSTEMATIC REVIEW AND META-ANALYSIS OF RANDOMIZED CONTROLLED TRIALS

**Authors:**

Karine A. Al Feghali, MD, MS ^1^*, Rami A. Ballout, BS ^2^, Assem M. Khamis, MD, MPH ^3^, Elie A. Akl, MD, PhD ^4^, Fady B.Geara, MD, PhD^1^

^1^Department of Radiation Oncology, American University of Beirut Medical Center, Beirut, Lebanon

^2^Faculty of Medicine, American University of Beirut, Beirut, Lebanon

^3^Department of Diagnostic Radiology, American University of Beirut Medical Center, Beirut, Lebanon

^4^Department of Internal Medicine, American University of Beirut Medical Center, Beirut, Lebanon

***Correspondence:**

Karine A. Al Feghali, MD, MS

Department of Radiation Oncology

American University of Beirut Medical Center

Beirut, Lebanon

karinefeghali@hotmail.com

# Supplementary Data

**1.1. Supplementary data 1: Search strategy**

- - 1. **Database: Ovid MEDLINE(R) <1946 to February Week 3 2014>**

**(The same search was run again until July 2016)**

1 randomized controlled trial.pt. (363020)

2 controlled clinical trial.pt. (87529)

3 randomized.ab. (263363)

4 placebo.ab. (142471)

5 drug therapy.fs. (1664948)

6 randomly.ab. (187940)

7 trial.ab. (271136)

8 groups.ab. (1212670)

9 1 or 2 or 3 or 4 or 5 or 6 or 7 or 8 (3117256)

10 humans.sh. (13184976)

11 9 and 10 (2549148)

12 exp Lung Neoplasms/ (168804)

13 exp Carcinoma, Bronchogenic/ (39136)

14 exp Bronchial Neoplasms/ (48792)

15 exp Pleural Neoplasms/ (10809)

16 ((lung* or pulmonary or bronch* or pleura*) adj2 (carcinoma* or cancer* or tumor* or tumour* or neoplas*)).mp. [mp=title, abstract, original title, name of substance word, subject heading word, keyword heading word, protocol supplementary concept word, rare disease supplementary concept word, unique identifier] (197526)

17 12 or 13 or 14 or 15 or 16 (200699)

18 exp Carcinoma, Large Cell/ (1838)

19 exp Adenocarcinoma/ (277518)

20 exp Carcinoma, Squamous Cell/ (101275)

21 (squamous adj cell adj4 (carcinoma* or cancer* or tumor* or tumour* or neoplasm*)).tw. (60223)

22 (large adj cell adj4 (carcinoma* or cancer* or tumor* or tumour* or neoplasm*)).tw. (3413)

23 adenocarcinoma*.tw. (89669)

24 (lung* or pulmonary or bronch* or pleura*).tw. (805435)

25 18 or 19 or 20 or 21 or 22 or 23 (394138)

26 24 and 25 (49473)

27 NSCLC.ti,ab. (16355)

28 exp Carcinoma, Non-Small-Cell Lung/ (30382)

29 (((non adj small) or nonsmall) adj3 ((lung* or pulmonary or bronch* or pleura*) adj3 (carcinoma* or cancer* or tumor* or tumour* or neoplas*))).mp. (35791)

30 29 or 26 or 27 or 28 (77115)

31 exp Carcinoma, Small Cell/ (16406)

32 SCLC.ti,ab. (4694)

33 ((lung* or pulmonary or bronch* or pleura*) adj3 (small adj2 (carcinoma* or cancer* or tumor* or tumour* or neoplas*))).mp. (33394)

34 31 or 32 or 33 (47045)

35 17 or 30 or 34 (213159)

36 35 not (34 not (34 and 30)) (201358)

37 exp Cranial Irradiation/ (4108)

38 pci.tw. (11374)

39 wbrt.tw. (698)

40 ((brain or crani* or head* or skull*) adj3 (radiotherap* or irradiat* or radiat*)).mp. (13019)

41 37 or 38 or 39 or 40 (25090)

42 11 and 36 and 41 (459)

**1.1.2. Database: EMBASE**

No.Query

Results

722

#38

#1 AND #31 AND #37

69,210

#37

#32 OR #33 OR #34 OR #35 OR #36

1,435

#36

wbrt:ab,ti

26,763

#35

pci:ab,ti

42,700

#34

(brain OR crani* OR head* OR skull*) NEAR/3 (radiotherap* OR irradiat* OR radiat*)

3,975

#33

'brain radiation'/exp

2,616

#32

'skull irradiation'/exp

305,525

#31

#30 NOT (#29 NOT (#29 AND #22))

321,793

#30

#7 OR #22 OR #29

80,199

#29

#23 OR #26 OR #27 OR #28

79,443

#28

lung* OR pulmonary OR bronch* OR pleura* AND small NEAR/2 (carcinoma* OR cancer* OR tumor* OR tumour* OR neoplas*)

6,769

#27

sclc:ab,ti

6,965

#26

#24 AND #25

1,816,340

#25

lung* OR pulmonary OR bronch* OR pleura*

9,490

#24

'small cell carcinoma'/exp

16,377

#23

'lung small cell cancer'/exp

117,949

#22

#16 OR #17 OR #18 OR #19 OR #20 OR #21

62,828

#21

non NEAR/1 small OR nonsmall AND (lung* OR pulmonary OR bronch* OR pleura*) NEAR/2 (carcinoma* OR cancer* OR tumor* OR tumour* OR neoplas*)

55,722

#20

'lung non small cell cancer'/exp

29,842

#19

nsclc:ab,ti

4,896

#18

'lung squamous cell carcinoma'/exp

16,194

#17

'lung adenocarcinoma'/exp

67,893

#16

#14 AND #15

1,816,340

#15

lung* OR pulmonary OR bronch* OR pleura*

327,145

#14

#8 OR #9 OR #10 OR #11 OR #12 OR #13

70,343

#13

'adenocarcinoma'/exp

171,272

#12

adenocarcinoma*

54,179

#11

large NEAR/1 cell AND (carcinoma* OR cancer* OR tumor* OR tumour* OR neoplasm*)

132,055

#10

squamous NEAR/1 cell AND (carcinoma* OR cancer* OR tumor* OR tumour* OR neoplasm*)

97,802

#9

'squamous cell carcinoma'/exp

3,047

#8

'large cell carcinoma'/exp

305,960

#7

#3 OR #4 OR #5 OR #6

305,960

#6

(lung* OR pulmonary OR bronch* OR pleura*) NEAR/2 (carcinoma* OR cancer* OR tumor* OR tumour* OR neoplas*)

2,368

#5

'pleura cancer'/exp

2,254

#4

'bronchus cancer'/exp

254,042

#3

'lung tumor'/exp

1,691,809

#1

'crossover procedure'/exp OR 'crossover procedure' OR 'double blind procedure'/exp OR 'double blind procedure' OR 'randomized controlled trial'/exp OR 'randomized controlled trial' OR 'single blind procedure'/exp OR 'single blind procedure' OR random* OR factorial* OR crossover* OR (cross AND over*) OR 'cross near/2 over' OR placebo* OR doubl* NEAR/1 blind* OR singl* NEAR/1 blind* OR assign* OR allocat* OR volunteer*

**1.1.3. Database: PubMed**

((((((((randomized controlled trial[pt]) OR controlled clinical trial[pt]) OR randomized[tiab]) OR placebo[tiab]) OR drug therapy[sh]) OR randomly[tiab]) OR trial[tiab]) OR groups[tiab]) AND ((((Lung Neoplasms) OR (Carcinoma, Bronchogenic) OR (Bronchial Neoplasms) OR (Pleural Neoplasms) OR (((lung* OR pulmonary OR bronch* OR pleura*) AND (carcinoma* OR cancer* OR tumor* OR tumour* OR neoplas*)))) OR ((((lung* OR pulmonary OR bronch* OR pleura*)) AND ((Carcinoma, Large Cell) OR (Adenocarcinoma) OR (Carcinoma, Squamous Cell) OR ((squamous AND cell AND (carcinoma* OR cancer* OR tumor* OR tumour* OR neoplasm*))) OR ((large AND cell AND (carcinoma* OR cancer* OR tumor* OR tumour* OR neoplasm*))) OR (adenocarcinoma*))) OR (NSCLC[tiab]) OR (Carcinoma, Non-Small-Cell Lung) OR ((((non AND small) OR nonsmall) AND ((lung* OR pulmonary OR bronch* OR pleura*) AND (carcinoma* OR cancer* OR tumor* OR tumour* OR neoplas*))))) OR ((Carcinoma, Small Cell) OR (SCLC[tiab]) OR (((lung* OR pulmonary OR bronch* OR pleura*) AND (small AND (carcinoma* OR cancer* OR tumor* OR tumour* OR neoplas*)))))) NOT (((Carcinoma, Small Cell) OR (SCLC[tiab]) OR (((lung* OR pulmonary OR bronch* OR pleura*) AND (small AND (carcinoma* OR cancer* OR tumor* OR tumour* OR neoplas*))))) NOT (((Carcinoma, Small Cell) OR (SCLC[tiab]) OR (((lung* OR pulmonary OR bronch* OR pleura*) AND (small AND (carcinoma* OR cancer* OR tumor* OR tumour* OR neoplas*))))) AND ((((lung* OR pulmonary OR bronch* OR pleura*)) AND ((Carcinoma, Large Cell) OR (Adenocarcinoma) OR (Carcinoma, Squamous Cell) OR ((squamous AND cell AND (carcinoma* OR cancer* OR tumor* OR tumour* OR neoplasm*))) OR ((large AND cell AND (carcinoma* OR cancer* OR tumor* OR tumour* OR neoplasm*))) OR (adenocarcinoma*))) OR (NSCLC[tiab]) OR (Carcinoma, Non-Small-Cell Lung) OR ((((non AND small) OR nonsmall) AND ((lung* OR pulmonary OR bronch* OR pleura*) AND (carcinoma* OR cancer* OR tumor* OR tumour* OR neoplas*)))))))) AND ((Cranial Irradiation) OR (pci[tiab]) OR (wbrt[tiab]) OR (((brain OR crani* OR head* OR skull*) AND (radiotherap* OR irradiat* OR radiat*))))

**1.2. Supplementary data 2: Methods used to derive hazard ratios (HR)**

Parmar et al. proposed three methods that allowed us to recalculate a HR and its standard error. First, the standard error (SE) was calculated indirectly in two studies (Li et al.^51^ and Gore et al.^48,49^) that reported the HR and its 95% CI based on this equation:

SE [ln(HR)] = $\frac{upper CI-lower CI}{2*1.96}$

Second, we used the number of observed events in the intervention (Or) and control arm (Oc), the total number of observed events (O), the total randomized number of patients in each arm (Rr, Rc), and the *p*-value of the log-rank test (if available) to recalculate the HR and its SE in three studies (Russel et al., Cox et al. and Miller et al.) per these equations:

ln(HR) = $\frac{O-E}{Vr}$ and var[ln(HR)]= 1/Vr

where: O – E = $\frac{\sqrt{O Rc Rr}}{Rc+Rr}X Z_{\left( 1-\frac{p}{2} \right)}$ and Vr = $\frac{Oc Or}{O}$

Third, when the trial report did not provide the hazard ratios (no number of events, total randomized number, or log-rank p-value was provided), and the only information available was a Kaplan-Meier (KM) curve, we used pixel coordinates to determine survival rates, working backwards and extracting data points from the KM curve. Umswasadi et al. did not report the *p*-value of log-rank test ^31^, so we estimated the HR through analyzing the KM curve using online software (WebPlotDigitizer, a web-based tool to extract data from plots) and the equations mentioned in Parmar’s study. Although The Cochrane Handbook does not address methods for data extraction from figures ^78^, a recently published paper has shown that extracting data from figures is more reliable than manual extraction ^79^. The second and third methods were tested on other studies (Li et al. ^51^ and Gore et al.^48,49^) that reported HR to compare between reported and calculated value of HR.

# Supplementary data 3: Ongoing trials

In order to determine whether our unanswered questions are being addressed, we searched four large clinical trial registries on December 10th, 2016 for any ongoing or completed trials relevant to our review for their potential inclusion in this manuscript or future versions of the manuscript. We searched the North American (<http://clinicaltrials.gov/>), European (<https://www.clinicaltrialsregister.eu>), World Health Organization (WHO) (<http://apps.who.int/trialsearch/>), and BMC (<http://www.isrctn.com/>) trial registries.

Of the seven trials captured by this search, one was completed with its results posted in October 2015 (NCT00048997) and its results are already included in this meta-analysis ^48–50^. A Chinese trial entitled “Prophylactic Cranial Irradiation (PCI) Versus no PCI in Non-Small Cell Lung Cancer After a Response to Chemotherapy” (NCT00745797) was opened in 2008 and terminated back in January 2014 due to slow accrual and lack of funding. Another trial from the Netherlands had a “completed” status in April 2015 (NCT01282437), with no published reports yet. This trial is interesting in that it randomized only stage III (IIIA and IIIB) patients to PCI or no PCI, and is examining development of symptomatic brain metastases as a primary endpoint, and time to development of neurological symptoms, side effects from PCI, and QOL/NCF (NCT01290809) as secondary endpoints. We marked this trial for tracking to follow up on its results as soon as they are available for it inclusion in future updates of our review. Two other trials had an “ongoing but not recruiting” status, a Chinese study NCT02448992: “Hippocampal-Sparing Prophylactic Cranial Irradiation in Pathologically Nodal Positive Non-Small-Cell Lung Cancer” and a Mexican study NCT01603849: “Prophylactic Cranial Irradiation in Patients With Lung Adenocarcinoma With High Risk of Brain Metastasis”, in January and February 2016 respectively. We also marked these two trials for tracking for future inclusion in updated versions of our review. It is interesting that one is trying to maximize the benefit-risk ratio by attempting to minimize the neurocognitive side effects of PCI, and the other is looking at a high-risk group, i.e. patients with a high-risk histology, i.e., adenocarcinoma. Of the two remaining trials, one had a “recruiting” status back in July 2010 (Chinese trial NCT01158170: Prophylactic Cranial Irradiation in Erlotinib/Gefitinib-responders With Non-small Cell Lung Cancer (NSCLC)) while the other had a “not yet recruiting” status in January 2010 (Korean trial NCT00955695: “A Randomized, Phase III Trial of Prophylactic Cranial Irradiation (PCI) in Patients With Advanced Non-small Cell Lung Cancer (NSCLC) Who Are Nonprogressive on Gefitinib or Erlotinib”. This trial is reserved for stage IIIB and IV NSCLC). Both trials have no reported results and their statuses on clinicaltrials.gov have not been updated since 2010. The Dutch (NCT01282437/ NCT01290809) and the Mexican (NCT01603849) ongoing studies reported above both address QOL and NCF, and might allow us in future versions of this review to perform a meta-analysis for QOL and NCF outcomes, by pooling their results with results from the trials by Li et al. ^53^, and Sun et al. ^50^, already included in the current review. The main issue with QOL/NCF outcomes is that different trials are using various instruments for assessment, which precludes pooling these results in a meta-analysis.
